# Effect of various postmortem processing times and blanching methods on quality of rainbow trout (*Oncorhynchus mykiss*) waste oil

**DOI:** 10.1002/fsn3.1171

**Published:** 2019-08-22

**Authors:** Forouzan Sabzipour, Mahmood Naseri, Sedigheh Babaei, Ahmad Imani

**Affiliations:** ^1^ Department of Natural Resources and Environmental Engineering, School of Agriculture Shiraz University Shiraz Fars Iran; ^2^ Department of Fisheries, Faculty of Natural Resources Urmia University Urmia Iran

**Keywords:** oil, oxidation, quality, valorization, viscera

## Abstract

Oil extracted from fish waste is considered as a value‐added product. The effect of postmortem processing times (0, 3, 6, and 9 hr) and blanching methods (sodium chloride, pH shift, and high temperature) on the extracted oil from rainbow trout viscera was studied. Blanching was applied six hours prior to oil extraction to counteract the effects of delayed processing time and increasing the oil stability. Autolysis by digestive enzymes is main culprit of higher contents of free fatty acids, lipid oxidation, saponified compound, and saturation degrees in case of postponed oil extraction. Results showed that PV was increased after pH shift and high temperature blanching, while there were no significant differences by using salt blanching. The lowest amount of TBA, AV, Totox, and saponification index was observed in salt blanched treatment. The colorimetric values including L*, b*, and whiteness index were decreased after pH shift, whereas redness was increased. Unfavorable coloration could be attributed to the lipid oxidation process that giving rise nonvolatile decomposition products with carbonyl groups. Our results indicated that salt blanching could reduce the effects of delayed processing time and lead to higher quality value‐added product from rainbow trout viscera.

## 1 INTRODUCTION

More than 50% of fish tissues including skin, fins, viscera, head, and backbone are discarded during various processing operations as "wastes" (Caruso, 2015). It has been estimated that near to 75% of the total fisheries product is discarded via fish processing industry (Olsen, Toppe, & Karunasagar, 2014). The wastes are good sources of industrially important constituents with numerous nutraceutical applications and functional properties (Ramakrishnan, Ghaly, Brooks, & Budge, 2013). Rainbow trout (*Oncorhynchus mykiss*) with roughly ~ 815 thousand tons’ production in 2016 (FAO, 2018) is a good source of recovering value‐added products from processing wastes since it would be a wise solution to environmental pollution and also provides economic benefits to the industry.

Peru and Chile, as the main fish oil producers, share about 52% and 13% of the total world fish oil production, while the share of Iceland and Norway is only 7% adequate to meet the whole Europe demand. With this in view, it is expected using fish processing wastes as alternative sources for fish oil would palliate fishing pressure on wild fish stocks for meal and oil production and contribute to environmentally viable aquaculture industry (Caruso, 2015). Fish by‐products and waste contribution to total fishmeal and fish oil production are close to 25 up to 35% and are expected to increase in future (Seafish, 2018).

Fish oil, as a biologically active and valuable compound, due to its highly unsaturated fatty acid contents of omega‐3 family, including eicosapentaenoic acid (EPA; 20:5n‐3) and docosahexaenoic acid (DHA; 22:6n‐3), is highly recommended for normal human/animal growth and development (Fiori et al., 2012). Facilitating the availability and increasing the consumption of marine lipids are mostly considered as a priority for healthcare authorities especially for nutritionally compromised poor people (Hao et al., 2015).

Via various operations including chemical and enzymatic processing, cooking, and pressing, it can be recovered from waste (Ramakrishnan et al., 2013). For instance, EPA and DHA were detected in the oil extracted from skin, spinal cord, head, and viscera of mackerel (*Rastrelliger kanagirta*) and rainbow trout (Fiori et al., 2012). However, high susceptibility to rancidity mainly due to various double carbon–carbon bonds present in fatty acid molecules may render the oil less valuable or even useless (Benjakul & Morrissey, 1997). Valorization of fish processing wastes, for instance extracting such valuable oil, is subject of recent studies (Carsuo, 2015; Fiori et al., 2012; Nascimento et al., 2015; Nazir, Diana, & Sayuti, 2017). Various extraction methods have been proposed to extract oil from seafood wastes (Cyprian et al., 2016; Deepika et al., 2014; Hao et al., 2015; Laila, Wahidu, & Tajul, 2014). One of the most common methods for maintaining food quality as well as controlling oxidation, enzymatic, chemical, and microbial activity is freezing (Anoosheh, Hosseini, & Mirsadeghi, 2015; Aubourg, 2001; Ghaly, Dave, Budge, & Brooks, 2010; Karungi, Byaruhanga, & Muyonga, 2004). Quality of final product is mainly dependent upon primary processing, temperature changes, postmortem freezing time, storage conditions, etc. (Aubourg, 2001; Boran, Karaçam, & Boran, 2006).

Surprisingly, the degradation rate of waste oil is more higher than the extracted oil from the fish tissue. Previous experiences have shown that within a few hours (<15 hr), oxidation products are heavily increased in oil extracted from fish waste due to the presence of digestive enzymes and oxidant agents (Erkan & Özden, 2006). When equipment for immediate freezing or processing is not available, fish and fish waste might remain at ambient temperature for hours upon capture accelerating its putrefaction and negatively affecting the final product quality (Rezaei, Hosseini, Langrudi, Safari, & Hosseini, 2008). Autolytic enzymes, especially digestive enzymes such as lipase, play a major role in fish decomposition upon death. The activity of lipase, after fish death, leads to lipid decomposition and increased free fatty acid content of the oil, which will facilitate oxidation, leaving unpleasant odor and taste with follow‐up discoloration (Aubourg, 2001).

Sodium chloride, pH change, or high temperature has been successfully used to improve the quality of products via eliminating or inhibiting enzymes activity (Ghaly et al., 2010; Omodara & Olaniyan, 2012). For instance, increasing salt concentration up to 30% at 55°C along with appropriate ambient pH could entirely stop the proteolytic activity in tuna splenic extract (Klomklao, Benjakul, & Visessanguan, 2004). Furthermore, in Indian anchovy (*Stolephorus indicus*), adding 25% sodium chloride reduced autolytic activity by 48% (Siringan, Raksakulthai, & Yongsawatdigul, 2006).

Due to tremendous quantity of cultured rainbow trout worldwide, the produced waste and their value‐added products are of great importance. Considering the economic importance and nutritional value of fish waste oil, quality preservation and shelf‐life enhancement of this perishable product are inevitable. Up to date, there is not any scientific publication regarding the effect of delayed processing and blanching methods on the waste oil quality. That's why, to determine the maximum possible delay time in processing, the quality of the oil extracted from the waste was evaluated at different freezing time points after gutting in the first trial. In the second trial, three various blanching methods including sodium chloride, lower ambient pH, and high temperature treatments (boiling water) were applied before oil extraction from viscera in order to find the appropriate blanching treatment to counteract the effects of delayed processing of the wastes and increase the stability of extracted oil.

## 2 MATERIALS AND METHODS

### 2.1 Trial 1

This experiment was set up to determine the maximum possible delay time before processing waste by means of various oil quality indices.

#### 2.1.1 Materials

All chemicals, reagents, and standards with premium quality were obtained from Merck (Merck).

#### 2.1.2 Fish viscera

Live rainbow trout were obtained from a local provider, Shiraz, Iran. All procedures were carried out according to Animal Ethics Committee Approval of Shiraz University. Fish were anesthetized by clove oil and euthanized by severing the spinal cord. Viscera was obtained and washed by cold distilled water. The viscera from different fish were pooled and divided into four parts (with three replicates). One part was immediately frozen at −28ºC, and the others were kept at room temperature (25ºC) for 3, 6, and 9 hr. before being frozen. After 24 hr of keeping at −28ºC, oil was extracted from frozen viscera and subjected to various quality control analyses.

#### 2.1.3 Oil extraction

The oil was extracted by solvent according to Bligh and Dyer (1959). In brief, about 50 g of fish waste was homogenized in a mixture of 200 ml chloroform, 200 ml methanol, and 150 ml distilled water. The mixture was filtered, and solvent was removed using rotary evaporator (Vacuum Rotary) at 40ºC and kept refrigerated until analyses in a dark screw‐topped bottle (Tavakoli, Naseri, Abedi, & Imani, 2018).

#### 2.1.4 Fish oil quality indices

Peroxide value (PV) was determined according to AOCS (Cd 8–53), in which PV is reported as meq of active oxygen per kg oil (AOCS, 1995). The thiobarbituric acid (TBA) value (mg malondialdehyde. kg^‐1^ oil) was measured according to Egan, Kirk, and Sawyer (1997). Free fatty acid (FFA) content was assessed following AOCS (Ca 5a ‐ 406) (AOCS, 1995) and expressed as percent of oleic acid. Anisidine value (AV) was determined using AOCS (Cd 18–90) (AOCS, 1995). Totox value was calculated by adding AV to twice the PV (Deepika et al., 2014). Saponification value (SV) and iodine value (IV) were also determined following AOCS (Cd 3–25 and 1b‐87, respectively) (AOCS, 1995).

#### 2.1.5 Color measurement

Color properties of the extracted oil were evaluated in a smart color analyzer (MAH, 3000 Series. IDME Co., Ltd.) based on CIE Lab System, in which L* denotes lightness, a* was the representative of redness or greenness, and b* indicated yellowness or blueness of the oil. The color difference (*∆E*) between the sample and the standard was calculated using ∆*E* = [(∆L*)^2^ + (∆a*)^2^ + (∆b*)^2^]^0.5^ equation (Plataniotis & Venetasanopoulos, 2000). ∆L* was the difference in brightness between a given sample and the standard, whereas ∆a* and ∆b* were the differences in the color coordinates a* and b*, respectively. The standard in this experiment was the control group, which was immediately frozen at −28ºC. In addition, the whiteness index (WI) was calculated according to Ojagh, Rezaei, Razavi, and Hosseini, (2010).

### 2.2 Trial 2

After determining the effect of various time intervals of postmortem processing time of fish viscera oil, the second trial was carried out to evaluate the effect of three different blanching methods on oil quality indices prior to extraction in order to find the most suitable method to counteract the delay in processing of viscera and increase oil stability.

#### 2.2.1 Blanching methods

Fish viscera were differently blanched using boiling water, salt, or pH shift. Blanching in boiling water (100°C) was done after packing viscera in polyethylene bags (Fernandez‐Segovia, Camacho, Martinez‐Navarrete, Escriche, & Chiralt, 2003). For salt treatments, the waste was dipped in saturated sodium chloride solution in a ratio of 4:1 (waste to salt) for 15 min (Siringan et al., 2006). pH change was done according to the method described by Siringan et al. (2006). In brief, viscera was immersed in the acidic buffer of 0.1 M sodium citrate and 0.2 M sodium phosphate (pH = 4) in a ratio of 6:1 (waste to acidic buffer) for 15 min (Siringan et al., 2006). Oil extraction was carried out immediately after blanching (BW) and 6 hrs after blanching (SBW). Chemical and physical parameters as quality indicators of the extracted oil were measured as above.

#### 2.2.2 Extracting enzymes

Fish waste was homogenized in 2% sodium chloride solution (1g waste in 5 ml of NaCl solution) using electric homogenizer (WIGGEN, D500). The supernatant was collected after centrifuging at 5,000 g for 30 min at 4°C (Hermle Z36HK).

#### 2.2.3 Enzyme assay

Lipase (E.C.3.1.1) activity was determined by hydrolysis of n‐nitrophenyl myristate. Each assay (0.5 ml) contained 0.53 mM n‐nitrophenyl myristate, 0.25 mM 2‐methoxyethanol, 5 mM sodium cholate, and 0.25 M Tris–HCl (pH 9.0). The reaction was terminated by adding 0.7 ml of acetone/n‐heptane (5:2, v/v) after incubating for 15 min at 25°C. The reaction mixture was vigorously mixed and centrifuged at 6,080 g for 2 min. The absorbance was read at 405 nm in the resulting lower aqueous layer. The extinction coefficient of n‐nitrophenol was 16,500 M^‐1^ cm^‐1^ L^‐1^. One unit of enzyme activity was defined as 1 µmol of n‐nitrophenol released per min (Iijima, Tanaka, & Ota, 1998). As substrate for trypsin (E.C.3.4.21.4), 1mM solution of N‐α‐benzoyldlarginine‐p‐nitroanilide (BAPNA) was prepared in 0.05 M Tris–HCl, pH 7.5, 0.02 M CaCl_2_ buffer and incubated at 37°C. Absorbance was read at 410 nm (Erlanger, Kokowsky, & Cohen, 1961).

Total soluble protein was detected by Bradford protein assay (Bradford, 1976), and enzyme activities were expressed as specific activity (U mg per protein).

### 2.3 Statistical analysis

Data analysis was carried using SPSS ver. 21 software. Shapiro–Wilk test of normality was used to determine whether the dataset was normally distributed. One‐way analysis of variance (ANOVA) was performed to check whether there were any differences in oil quality parameters regarding different intervals before viscera processing or blanching treatments. Tukey's honestly significant difference test (HSD) was used for *post hoc* analyses. Dunnett *T*_3_ test was used when homoscedasticity of variances was violated. Paired‐samples *t* test was also used to look for any differences between two different time points when samples received blanching treatments.

## 3 RESULTS AND DISCUSSION

### 3.1 Trial 1

#### 3.1.1 Peroxide value and thiobarbituric acid test

Peroxide index (PV) determines the concentration of peroxides and hydroperoxides formed during the initial stage of fats and oil oxidation. In the case of prolonged oxidative rancidity, such primary oxidation products decompose to form aldehydes and ketones, which are measured by TBA test (Aubourg, 2001). PV and TBA values measured in the oil extracted from frozen waste at different time points are shown in Table 1. They present an increasing trend. Rezaei et al. (2008) found that up to 8 hr delay in postharvest icing does not affect fillet PV, while results in shortened shelf life of 1–3 days. They reported that PV of the fish fillet is lower than 1 meq O_2_/kg fat. Since PV is limited to the initial stages of oxidation, the difference in PVs became significant at the end of the storage period (Jeon & Shahidi, 2002; Rezaei et al., 2008; Woyewoda, Shaw, Ke, & Burns, 1986). We find that PV of extracted oil is in the range those reported by others (Faraji‐dana & Gharehkhani, 2016; Norziah, Nuraini, & Lee, 2009; Yin, Solval, Huang, Bechtel, & Sathivel, 2011). In this study, it has been shown that PV immediately exceeded over 10 meq O_2_/kg in oil extracted from trout waste during the first 3 hr. However, a rise in TBA value is only observed in longer delays for viscera freezing, possibly due to increased availability of iron and other prooxidants in gut and/or formation of secondary oxidation products as a result of hydroperoxide decomposition (Anoosheh et al., 2015; Aubourg, 2001).

**Table 1:** The effect of delayed freezing on quality parameters of oil extracted from rainbow trout wastes

Freezing delay time (hour)	Indices of oil spoilage						
	Peroxide value (meq O_2_/kg oil)	Thiobarbituric acid (mg malondialdehyde/kg oil)	Free fatty acids (oleic acid %)	Anisidine value	Saponification value (mg KOH/g oil)	Iodin value (g I_2_. 100/g)	TOTOX
0	9.43 ± 0.10^c^	0.11 ± 0.01^c^	0.19 ± 0.00^d^	0.51 ± 0.00^c^	149 ± 4.9^d^	46.12 ± 0.72^a^	19.3 ± 0.20^d^
3	14.51 ± 0.47^b^	0.12 ± 0.01^c^	0.28 ± 0.01^c^	0.56 ± 0.01^b^	177 ± 5.8^c^	43.43 ± 2.27^ab^	29.5 ± 0.94^c^
6	16.34 ± 0.33^a^	0.16 ± 0.01^b^	0.31 ± 0.00^b^	0.83 ± 0.00^a^	197 ± 11.8^b^	36.09 ± 4.2^c^	33.5 ± 0.45^a^
9	15.46 ± 0.40^a^	0.19 ± 0.02^a^	0.33 ± 0.01^a^	0.55 ± 0.01^b^	220 ± 3.4^a^	37.35 ± 2.74^bc^	31.4 ± 0.80^b^

Note

Different superscripts in each column show statistical differences among different freezing time delays in viscera oil extraction (*p* < .05) (one‐way ANOVA). Values are shown as means ± *SD*.

Thiobarbituric acid contents of all delayed frozen samples vary from 1.1 to 1.9 (mg malondialdehyde/kg oil). The mean values of TBA are considerably lower than the allowable limit (3 mg/kg) recommended for fish products by Al‐Kahtani et al. (1996). Samples with a longer delay time for freezing present the highest mean levels (1.9 mg malondialdehyde/kg oil). The mean values of TBA content of extracted oil were considerably higher than those values reported in fish fillet by Abedi, Naseri, Ghanbarian, and Vazirzadeh, (2016) and Tavakoli et al. (2018); meanwhile, they are similar to those reported by Nazir et al. (2017) for tuna waste oils obtained using various extraction methods. Increasing TBA values during storage time are also reported in the oil extracted from Caspian white fish (*Rutilus frisii kutum*) and Nile perch (*Lates niloticus*) when freezing delay time becomes longer (Karungi et al., 2004).

#### 3.1.2 Free fatty acids

Lipases and phospholipases hydrolyze triacylglycerol to free fatty acids (FFAs) and glycerol during storage time (Aubourg, 2001). Increased free fatty acid content results in faster oxidation rate and undesirable off‐flavor development (Abedi et al., 2016). Karungi et al. (2004) assess FFA content of Nile perch frozen with 0‐, 3‐, and 6‐hr delay and report higher FFA content is associated with extended freezing time delays. In this study, FFA content of oil extracted from the waste with longer delay in freezing is high (Table 1), further confirming the efficiency of cold storage in preventing hydrolytic degradation in comparison with ambient temperature storage.

#### 3.1.3 Anisidine and Totox values

Primary oxidative products decompose and give rise to secondary oxidative products during processing and preservation. The AV determines the secondary oxidation products (especially α, β‐unsaturated aldehydes). Total oxidation value (Totox) is indicative of various compounds including hydroperoxides, aldehydes, and ketones, products of polyunsaturated fatty acid (PUFA) breakdown under pro‐oxidative condition (e.g., high temperatures, oxygen, metal compounds, and light) (Rubio‐Rodríguez et al., 2012). AV and Totox values of the oil extracted from waste frozen at different time points are shown in Table 1. In comparison with the control group immediately frozen after dissection, AV and Totox values of oil extracted from those viscera frozen 3 and 6 hr after fish dissection are significantly increased. However, the values are lower in samples with 9 hr’ delay in freezing time. Aubourg (1999) shows that oxidation products might react to other fish tissue constituents rendering primary or secondary products’ content an inaccurate measure to assess the degree of damage induced by delayed processing. According to the formula used to calculate Totox value (i.e., AV + 2PV), any reduction in primary oxidative products’ content toward the end of the storage period (9 hr) and the subsequent reaction with amine‐containing compounds to form fluorescent products (end products of lipid deterioration; Aubourg, 2001) might result in lower Totox value.

#### 3.1.4 Saponification and iodine values

Saponification value quantifies the amount of potassium hydroxide required for saponifying 1g of fat (Boran et al., 2006), while IV determines the relative degree of unsaturation (double bonds) in fatty acids (Nazir et al., 2017). Our results showed that SV increases as delay in viscera freezing increased, in contrast, IV significantly decreases. These observations are in agreement with previous study on quality of oil extracted from marlin by Boran et al. (2006). Regarding the relationship between SV and molecular weight of oil, it is conceivable that fatty acids with shorter chains would have higher SV (Nazir et al., 2017) mainly due to the presence of large numbers of functional carboxyl groups per mass of a fat. Higher SVs of oils extracted from trout wastes with increased delay in freezing time would be rational since final oxidation products, namely aldehydes and ketones, would result in higher TBA content of oil (Boran et al., 2006). Increased delay time in freezing the wastes would reduce the quality of extracted oil and negatively affect the degree of unsaturation resulting in decreased IV of extracted oil.

#### 3.1.5 Oil color change

The color of foods is an important characteristic determining consumer perception of product freshness (Fernandez‐Segovia et al., 2003). As delays in freezing time increase, the colorimetric values including L* (brightness) and b* (yellowness) of the extracted oil significantly decrease, whereas the value of a* (redness) increases (Table 2). Shabanpour et al. (2015) show that oxidation of unsaturated fatty acids (because of longer delays) is the main reason for increased redness and decreased brightness/yellowness indices of oil. Moreover, oil extracted from viscera with longer delay time before freezing presents significantly lower whiteness index (WI) indicating implying lower oil transparency. Color difference with reference to controls (∆E) shows a progressive increase correlated with increasing delay time in waste processing. Increasing the delay time in waste freezing reduces the quality of the extracted oil (significantly increase in PV, TBA, Totox, AV, FFA contents, and SV) and negatively affects the degree of unsaturation as previous reported by Zolfaghari, Shabanpour, and Falahzadeh (2011) and Fernandez‐Segovia et al. (2003). They attribute such color changes to formation of carbonyl compounds, which are produced during storage as results of lipid oxidation product. Our results reveal that for premium quality oil viscera should be immediately subjected to oil extraction or must be frozen; otherwise, even three hours of delay in waste freezing would result in lower quality oil.

**Table 2:** The effect of delayed freezing time on color indices of oil extracted from rainbow trout wastes

Freezing delay time (hours)	L*	a*	b*	*E	WI
0	33.69 ± 0.33^a^	−0.68 ± 0.22^c^	20.75 ± 0.20^a^	–	30.52 ± 0.26^a^
3	32.14 ± 0.10^b^	1.13 ± 0.44^b^	19.83 ± 0.08^b^	2.57^c^	29.29 ± 0.08^b^
6	29.37 ± 0.23^c^	1.22 ± 0.14^b^	18.11 ± 0.14^c^	5.41^b^	27.08 ± 0.19^c^
9	27.28 ± 0.18^d^	1.76 ± 0.29^a^	16.92 ± 0.16^d^	7.86^a^	25.32 ± 0.18^d^

Note

Different superscripts in each column show statistical differences among different freezing time delays in viscera oil extraction (*p* < .05) (one‐way ANOVA). Values are shown as means ± *SD*.

### 3.2 Trial 2

#### 3.2.1 Effect of different blanching methods on quality of oil from fish waste

Temperature fluctuation, delayed freezing, and inappropriate storage conditions can accelerate fish spoilage and influence properties of compounds produced from by‐products. On fishing vessels lacking on‐board freezers, spoilage is inevitable (Ersoy, Aksan, & Özeren, 2008; Rezaei et al., 2008). Due to severe autolytic activity of digestive enzymes, the quality of waste oil is reduced upon fish death. Fish freezing can retard spoilage by reducing enzymes activity of oxidation and microbial growth. Inactivating digestive enzymes could lead to maintain quality of fish by‐products for producing valuable products such as the waste extracted oil (Ghaly et al., 2010). Therefore, the effect of different blanching treatments six hours before oil extraction along with the effect of such methods on oil quality per se is studied.

#### 3.2.2 Peroxide value and thiobarbituric acid test

PV changes of the extracted oil from blanched waste (control group subjected to oil extraction immediately after catch) and stored blanched waste are shown in Table 3. Using heat treatment or pH shift results in increased PV of viscera oil (*p* ≤ .05), while there are no significant differences between salting method and control group (*p* > .05).

**Table 3:** The effect of different blanching methods on quality parameters of oil extracted from Rainbow trout wastes

Indices of oil spoilage		Blanching methods			
		Control	Boiling water	Salt	pH shift
Peroxide value (meq O_2_/kg oil)	BW	4.42 ± 0.38^Cb^	20.8 ± 0.08^Ba^	4.08 ± 0.23^Cb^	76.7 ± 2.04^Ab^
	SBW	16.7 ± 0.3^BCa^	21.5 ± 1.1^Ba^	11.5 ± 0.38^Ca^	133 ± 6.1^Aa^
Thiobarbituric acid (mg malondialdehyde/kg oil)	BW	1. 42 ± 0.002 ^Bb^	1. 67 ± 0.001 ^Ab^	1. 33 ± 0.003 ^Cb^	1. 7 ± 0.001^Ab^
	SBW	2. 43 ± 0.001^Ba^	2. 41 ± 0.002 ^Ba^	2. 06 ± 0.01^Ca^	3. 18 ± 0.001^Aa^
Free fatty acids (oleic acid %)	BW	0.323 ± 0.003^Bb^	0.293 ± 0.005^Cb^	0.314 ± 0.007^Bb^	0.346 ± 0.01^Ab^
	SBW	0.623 ± 0.015^Ba^	0.408 ± 0.011^Da^	0.532 ± 0.015^Ca^	0.698 ± 0.02^Aa^
Anisidine value	BW	0.433 ± 0.01^Cb^	2.36 ± 0.19^Ba^	0.27 ± 0.01^Cb^	3.26 ± 0.07^Aa^
	SBW	1.19 ± 0.08^BCa^	2.52 ± 0.13^Ba^	0.76 ± 0.02^Ca^	3.34 ± 0.11^Aa^
Saponification value (mg KOH/g oil)	BW	124 ± 15^Db^	284 ± 3.2^Ba^	194 ± 35^Ca^	444 ± 22^Aa^
	SBW	290 ± 1.8^Ba^	311 ± 14^Ba^	218 ± 19.5^Ca^	450 ± 29^Aa^
Iodin value (g I_2_. 100/g)	BW	46.86 ± 1.43^Aa^	38.09 ± 2.54^Aa^	38.04 ± 1.39^Aa^	33.83 ± 1.47^Ba^
	SBW	37.11 ± 2.72^Ab^	37.83 ± 0.60^Aa^	36.63 ± 2.55^Aa^	25.98 ± 2.35^Bb^
TOTOX	BW	9.28 ± 1.8^Ca^	43.96 ± 1.4^Ba^	8.43 ± 1.5^Ca^	156.7 ± 5.5^Aa^
	SBW	34.6 ± 0.51^Ca^	45.7 ± 2.3^Ba^	23.8 ± 0.77^Da^	268 ± 12.3^Aa^

Note

Control denotes nonblanched viscera, BW means oil immediately extracted from blanched or nonblanched waste, and SBW represents oil extracted from blanched or nonblanched waste after six hours of room temperature storage. Capital letters indicate statistical differences among various blanching methods, and small letters indicate statistical differences in oil quality indices of oil at different storage time (0 and 6 hr) (*p* < .05) Values are shown as means ± *SD*.

In stored blanched waste, the lowest PV is measured in salt blanched samples, but the increase of PV in those samples treated with boiling water was lower than other treatments. According to Aubourg (2001), Naseri, Rezaei, Moieni, Hosseni, and Eskandari (2010) and Abedi et al. (2016), PV is not an appropriate tool for evaluating quality differences during heating treatments, since during heating treatment hydroperoxides are thermally decomposed and give rise to secondary lipid oxidation products. It seems that blanching with salt could reduce the unfavorable changes during storage time and more effective in oxidation inhibition.

Thiobarbituric acid is one of the most important indices of oil quality. Determining malondialdehyde content is considered an appropriate measure of detecting secondary lipid oxidation products (Naseri et al., 2010). Thiobarbituric acid contents of extracted oil in various blanching treatments are shown in Table 3. Higher contents of secondary lipid oxidation products in sample treated with pH and boiling water indicate that oil rancidity extensively occurs during viscera blanching, while TBA index of salt‐treated viscera is the lowest (2.06 6 ± 0.01). Strong blanching treatments and presence of oxidation catalysts in viscera can intensify nonenzymatic lipid oxidation and hydrolysis and therefore result in nutrient loss. In stored blanched viscera (SBW), significant increase in TBA index is recorded; however, TBA increase of pH change and boiling water‐treated samples is higher than other groups (*p* ≤ .05). Our results are somehow indicative of inhibitory effect of salt blanching on oxidative enzymes previously reported by Reddi, Constantinides, and Dymsza (1972), Noori Hashem Abad, Hosseinipour, and Ojagh (2013) and Ghaly et al. (2010). For instance, Reddi et al. (1972) show that salt would result in reduced autolytic enzyme activity in winter flounder (*Pseudopleuronectes americanus*). Similarly, Anoosheh et al. (2015). Noori Hashem Abad et al. (2013) and Ghaly et al. (2010) report that sodium chloride solution decreases the activity of autolytic enzymes, especially lipase, and thereby reduces the formation of free fatty acids (oxidation‐sensitive products) and secondary oxidation products.

#### 3.2.3 Free fatty acids

Free fatty acid content is a proper indicator of enzymatic lipolysis (Aubourg, 1999). Free fatty acid contents of viscera oils are shown in Table 3. Free fatty acid contents of oil extracted from pH‐treated viscera are increased. During pH shift treatment, breakdown of high‐molecular‐weight lipids (triglycerides and phospholipids) would be likely occurred (Rai, Swapna, Bhaskar, Halami, & Sachindra, 2010). The FFA content of oil from salt‐treated viscera is not significantly different from that of control samples (*p* > .05). However, FFA content of oil is decreased after boiling water treatment. Such a decrease is attributable to loss of volatile free fatty acids during thermal processes leading to decreased FFA content (Aubourg, 2001; Naseri et al., 2010). In addition, decrease in FFA content after boiling water treatment might be due to deactivation of enzymes.

Comparison of FFA contents of oil immediately obtained from blanched waste and stored blanched waste (all methods) shows that the storage process leads to a significant increase in FFA content. This result is in agreement with those of Abedi et al. (2016) and Tavakoli et al. (2018). The highest increase in Free fatty acid is recorded in oil from stored pH shift‐treated viscera (0.698 ± 0.023). It seems nonenzymatic hydrolysis starts and propagates during storage and gives rise to increased free fatty acid content.

#### 3.2.4 Anisidine and Totox values

As presented in Table 3, AVs of viscera oils from boiling water and pH shift treatments are significantly increased. However, AV of control and salt blanched samples does not significantly differ (*p* > .05).

Measurement of secondary lipid oxidation product content is an appropriate quality parameter to evaluate the chemical changes from various processing treatments (Tavakoli et al., 2018). In this sense, carbonyl formation during blanching is widely used (Binsi et al., 2014; Cerretani, Bendini, Rodriguez‐Estrada, Vittadini, & Chiavaro, 2009; Cyprian et al., 2016). According to Table 3, storing blanched viscera results in quality loss. AV of oil from control group does not significantly differ from those of salt and boiling water blanched samples (*p* > .05). Noori Hashem Abad et al. (2013) report that salt treatment significantly reduces the activity of the lipase extracted from rainbow trout viscera. Decreased lipase activity results in reduced lipolysis and formation of FFA with subsequent reduction of oxidation rate and decreased peroxide, anisidine, and Totox values (Ghaly et al., 2010; Noori Hashem Abad et al., 2013). Totox value indicates the initial and later stages of oxidation of extracted oil and provides a reliable estimation of the progressive oxidative deterioration of final products. Totox values of pH shift‐ and boiling water‐treated viscera increase throughout the processing (Table 3). However, oil from salt‐treated viscera shows significantly lower Totox values (*p* ≥ .05). Moreover, Totox values of each treatment during storage period significantly differ (*p* ≥ .05). Acid‐treated samples show significantly higher Totox value in comparison with oil obtained from boiling water‐treated viscera. However, salt blanching protects oil from oxidative rancidity (Table 3). Our results reveal that the overall oxidation of waste oil is hampered by salt blanching.

#### 3.2.5 Saponification and iodine values

Effects of different treatments on SVs of extracted oil from trout viscera are shown in Table 3. Initial SV of oil is 124 mg KOH/g oil and blanching results in SV of 284, 194, and 444 mg KOH/g oil after boiling water, salt, and acid treatments, respectively. The pH shift treatment leads to the highest increase in SV mainly attributable to formation of the final lipid oxidation products (aldehydes and ketones). Simultaneously, the highest FFA content and TBA value in oil recovered after pH shift treatment indicate decreased lipid stability. However, salt and boiling water blanching would impede the fatty acid chain decomposition during storage of blanched viscera. In addition, the highest SV is detected in stored pH shift blanched viscera (SBW) (450 ± 29, *p* < .05) and the lowest SV belongs to stored salt blanched samples (218 ± 19.5). However, except for control group sample, alternations in SV of stored blanched samples (all treatments) were not statistically significant (*p* > .05). Similarly, improved quality of final products via eliminating or inhibiting enzymatic activity using blanching treatment is reported by others (Ghaly et al., 2010; Nazir et al., 2017; Omodara & Olaniyan, 2012).

Blanching, especially pH change solution treatment, results in decreased IV of oil from trout viscera (*p* < .05). IV of oil extracted from stored nonblanched and pH change blanched samples is decreased, while salt and boiling water treatments do not significantly affect IV of the extracted oil (*p* > .05). In this context, decreased IV could be explained by increase in PV, TBA, FFA, AV, Totox, and SV.

#### 3.2.6 Oil color change

The effect of different treatments on L* (brightness), a* (redness), b* (yellowness), and ΔE* (color difference) of the extracted oil is shown in Table 4. The highest L* and WI in extracted oil are belonged to salt blanched viscera. In contrast, decreased WI and L* and increased a* are observed in pH shift‐treated waste. At the same time, higher Totox value of oil from pH shift‐treated viscera also confirms incremental redness index. Shabanpour et al. (2015) believe that reduced lightness and increased redness are indicators of reduced oil quality and extensive oxidative degradation of fatty acids. Color difference with respect to the control group (∆E) shows a significant difference to the extent that it is the highest for pH shift blanching treatment (*p* < .05). Comparing the color indices in oil extracted from treated waste at different times (0 and 6 hr after blanching) shows a significant increase in a* value. The highest increase is recorded in the control samples (nonblanched viscera). Similarly, ΔE* is also increased in stored blanched samples. This observation can be explained by the interaction between the final products of lipid oxidation and amine‐containing moieties producing opacity and color difference with respect to control (Aubourg, 2001). Delayed extraction reduced whiteness index of nonblanched samples, whereas no significant differences in whiteness index are observed in various blanched treatments.

**Table 4:** The effect of different blanching methods on color indices of oil extracted from rainbow trout wastes

Color of oil		Blanching methods			
		Control	Boiling water	Salt	pH shift
L*	BW	36.5 ± 0.35^Ba^	33.7 ± 0.43^Ca^	41.7 ± 0.19^Aa^	15.3 ± 0.25^Db^
	SBW	26.5 ± 0.31^Bb^	33.4 ± 0.34^Aa^	17.6 ± 0.29^Db^	21.1 ± 0.34^Ca^
a*	BW	−2.27 ± 0.39^Cb^	0.13 ± 0.56^Bb^	−1.85 ± 0.32^Cb^	7.84 ± 0.16^Ab^
	SBW	8.53 ± 0.19^Ba^	5.71 ± 0.58^Ca^	9.61 ± 0.25^Aa^	8.98 ± 0.24^Aab^
b*	BW	22.4 ± 0.2^Ba^	20.8 ± 0.2^Ca^	25.6 ± 0.13^Aa^	9.7 ± 0.37^Db^
	SBW	16.4 ± 0.19^Bb^	20.8 ± 0.34^Aa^	11.05 ± 0.1^Db^	13.1 ± 0.21 ^Ca^
*∆*E*	BW	–	4.02^Cb^	6.08^Bb^	26.7^Ab^
	SBW	–	6.08^Ca^	9.05^Ba^	28.2^Aa^
WI	BW	32.6 ± 0.25^Ba^	30.5 ± 0.33^Ca^	36.2 ± 0.12^Aa^	14.4 ± 0.27^Da^
	SBW	24.2 ± 0.25^Bb^	30.4 ± 0.38^Aa^	36.4 ± 0.29^Aa^	14.5 ± 0.32^Ca^

Note

Control denotes nonblanched viscera, BW means oil immediately extracted from blanched or nonblanched waste, and SBW represents oil extracted from blanched or nonblanched waste after six hours of room temperature storage. Capital letters indicate statistical differences among various blanching methods, and small letters indicate statistical differences in oil quality indices of oil at different storage time (0 and 6 hr) (*p* < .05) Values are shown as means ± *SD*.

#### 3.2.7 Lipase and trypsin activities

**Figure 1:**
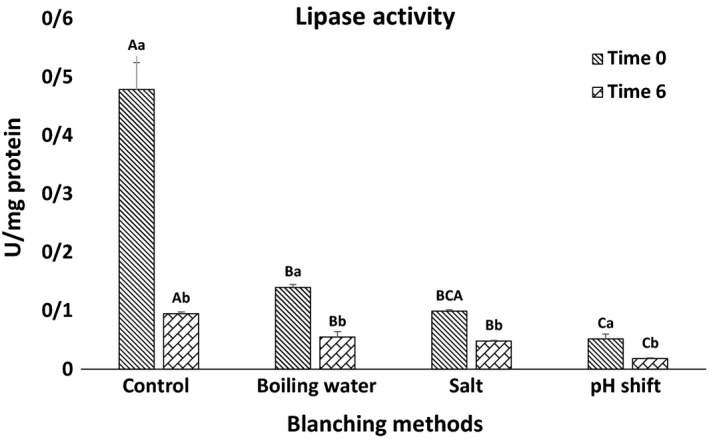
Specific activity (U. mg/protein) of lipase in Rainbow trout wastes in different times after blanching (0 and 6 hr). Time 0 means trypsin activity measured from blanched viscera, and time 6 represents evaluated lipase activity from blanched viscera after six hours of room temperature storage. Capital letters indicate statistical differences between blanching methods in every time point, and small letters indicate statistical differences in trypsin activity in two times (0 and 6 hr) (*p* < .05) Values are shown as means ± *SD*

**Figure 2:**
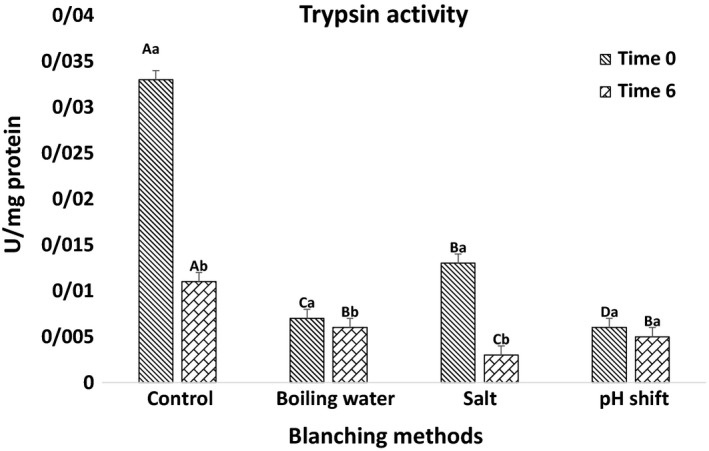
Specific activity (U. mg/protein) of trypsin in Rainbow trout wastes in different times after blanching (0 and 6 hr). Time 0 means trypsin activity measured from blanched viscera, and time 6 represents evaluated trypsin activity from blanched viscera after six hours of room temperature storage. Capital letters indicate statistical differences between blanching methods in every time point, and small letters indicate statistical differences in trypsin activity in two times (0 and 6 hr) (*p* < .05) Values are shown as means ± *SD*

Various digestive enzymes, namely lipases and proteases, are actively present in fish gut, especially pyloric ceca and intestine (Deguara, Jauncey, & Agius, 2003). The effect of different treatments on the activity of lipase and trypsin is shown in Figures 1 and 2, respectively. Activities of lipase and trypsin are significantly lower in the oil extracted immediately or six hours after treatments in comparison with nonblanched viscera. Among treatments, the highest and lowest activities are recorded in salt and pH shift treatments, respectively. According to Borlongan (1990), increased temperature denatures proteins, thereby results in reduced enzyme activity. Moreover, similar to our results Siringan et al. (2006) report that using table salt in Anchovy reduces activity of autolytic enzymes. Zamani (2016) shows reduced trypsin activity of the Caspian brown trout fry because of including NaCl and some metal ions (Mn^2+,^ Cu^2+^, Ba^2+^, Co^2+^, Zn^2+^, Fe^2+^, and Al^3+^).

## 4 CONCLUSION

Any delays in freezing time (0, 3, 6, and 9 hr) negatively affect the quality of extracted oil from rainbow trout viscera. PV immediately exceeds allowable limit during the first 3 hr. Unsaturation value (IV) is progressively reduced concomitant with increasing delay in freezing time. In the second trial, in order to reduce the effects of delayed processing on oil quality, three different blanching methods (sodium chloride, pH change, and high temperature) are applied to waste six hours before oil extraction. Salt treatment in general does not alter the oil quality and hampers formation of oxidation products. Chemical and color indices also show that good quality oil would be obtained from salt blanched waste even after a long delay in viscera oil extraction (i.e., 6 hr). In conclusion, the delay allowed for freezing rainbow trout viscera should not exceed three hours. Furthermore, salt treatment, as opposed to pH shift treatment, would yield better quality oil even after six hours of delay in extracting viscera oil. Such valorized product shows a possibility to be used for aquafeed production or even as a potential source of bioactive compounds for human nutrition.

## CONFLICT OF INTEREST

The authors have no financial or scientific conflict of interest with regard to the research described in this manuscript.

## ETHICAL APPROVAL

This study was approved by the Institutional Review Board of Shiraz University.

## INFORMED CONSENT

Written informed consent was obtained from all study participants.
